# Zinc Homeostasis in Platelet-Related Diseases

**DOI:** 10.3390/ijms20215258

**Published:** 2019-10-23

**Authors:** Elmina Mammadova-Bach, Attila Braun

**Affiliations:** 1 University Hospital and Rudolf Virchow Center, University of Würzburg, 97080 Würzburg, Germany; 2 Walther-Straub-Institute for Pharmacology and Toxicology, Ludwig-Maximilians University Munich, German Center for Lung Research, 80336 Munich, Germany

**Keywords:** Zinc, platelets, hemostasis, thrombosis, ischemic stroke, storage-pool diseases

## Abstract

Zn^2+^ deficiency in the human population is frequent in underdeveloped countries. Worldwide, approximatively 2 billion people consume Zn^2+^-deficient diets, accounting for 1–4% of deaths each year, mainly in infants with a compromised immune system. Depending on the severity of Zn^2+^ deficiency, clinical symptoms are associated with impaired wound healing, alopecia, diarrhea, poor growth, dysfunction of the immune and nervous system with congenital abnormalities and bleeding disorders. Poor nutritional Zn^2+^ status in patients with metastatic squamous cell carcinoma or with advanced non-Hodgkin lymphoma, was accompanied by cutaneous bleeding and platelet dysfunction. Forcing Zn^2+^ uptake in the gut using different nutritional supplementation of Zn^2+^ could ameliorate many of these pathological symptoms in humans. Feeding adult rodents with a low Zn^2+^ diet caused poor platelet aggregation and increased bleeding tendency, thereby attracting great scientific interest in investigating the role of Zn^2+^ in hemostasis. Storage protein metallothionein maintains or releases Zn^2+^ in the cytoplasm, and the dynamic change of this cytoplasmic Zn^2+^ pool is regulated by the redox status of the cell. An increase of labile Zn^2+^ pool can be toxic for the cells, and therefore cytoplasmic Zn^2+^ levels are tightly regulated by several Zn^2+^ transporters located on the cell surface and also on the intracellular membrane of Zn^2+^ storage organelles, such as secretory vesicles, endoplasmic reticulum or Golgi apparatus. Although Zn^2+^ is a critical cofactor for more than 2000 transcription factors and 300 enzymes, regulating cell differentiation, proliferation, and basic metabolic functions of the cells, the molecular mechanisms of Zn^2+^ transport and the physiological role of Zn^2+^ store in megakaryocyte and platelet function remain elusive. In this review, we summarize the contribution of extracellular or intracellular Zn^2+^ to megakaryocyte and platelet function and discuss the consequences of dysregulated Zn^2+^ homeostasis in platelet-related diseases by focusing on thrombosis, ischemic stroke and storage pool diseases.

## 1. Introduction

Zinc (Zn^2+^) is the second most important trace metal in the body. Zn^2+^ enters into the organism through dietary consummation and plays a critical role in many biological processes including development, proliferation, differentiation, cell metabolism, insulin synthesis and secretion, immune function, regulation of DNA synthesis and genomic stability [1,2]. In humans, daily Zn^2+^ ingestion can point to 14–30 mg/kg. The body absorbs 20–40% of Zn^2+^ in the gut, mainly in the jejunum through the enterocytes, and residual Zn^2+^ is excreted. Zn^2+^ is mainly stored in skeletal muscle (60%) and bone (30%), and only a small fraction of body Zn^2+^ is circulating in the blood at a concentration of 10–20 µM [3]. The major part of blood plasma-resident Zn^2+^ is covalently linked to albumin (75–85%) and β2-macroglobulin (10–20%) and a minor part (2%) can bind to amino acids, organic anions, histidine, cysteine and citrate. Therefore, only a small fraction of Zn^2+^ exists as free labile form (0.1–2 µM) which can be taken up by blood cells, also including endothelial cells and platelets [4,5,6]. Most of the cells can uptake Zn^2+^ through receptor-mediated endocytosis or other Zn^2+^ transport mechanisms [7,8].

Zn^2+^ has an important catalytic function by stabilizing negative charges in biochemical reactions in metabolic enzymes. Cellular Zn^2+^ uptake can increase the activity of several enzymes, such as mitogen-activated protein kinases (MAPKs), caspases and metalloproteinases (MMPs), [1,9,10,11,12]. Many of secretory cells, such as pancreatic β-cells, neutrophils and platelets, can uptake, store and release a significant amount of Zn^2+^, regulating diverse Zn^2+^-dependent intracellular and extracellular pathways [3,8,13,14,15,16,17,18,19]. Zn^2+^ is also responsible for maintaining the protein structure in zinc-finger transcription factors and LIM domain proteins, thus Zn^2+^ can be coordinated by oxygen, sulfur and nitrogen atoms of the polypeptide chain [20].

## 2. Zn^2+^ Homeostasis in Megakaryocytes and Platelets

The amount of metabolically active Zn^2+^ is very limited in the cell due to its cytotoxicity, therefore the majority of Zn^2+^ is associated to cytoplasmic proteins or transported and stored in intracellular organelles [1,3]. In mammalian cells, Zn^2+^ is mainly located in the nucleus, cytoplasm, endoplasmic reticulum, Golgi apparatus, vesicles and secretory granules. In the cytoplasm, Zn^2+^ is sequestered by metallothioneins (MTs), which are small cysteine-rich proteins, complexing up to seven Zn^2+^ ions [3]. Depending on the type and activated state of cells, approximatively 5–15% of cytoplasmic Zn^2+^ can bind to MTs [3]. When the sulfur group of MTs is oxidized, Zn^2+^ cannot bind MTs, thereby depleting MT-dependent Zn^2+^ store in the cytoplasm. Conversely, enhanced Zn^2+^ uptake induces MT gene expression by metal transcription factor 1 (MTF-1), thereby increasing the Zn^2+^ storage capacity of MTs inside the cell [3]. In human platelets, several MTs were detected and the amount of MT is approximatively 40 µg/10^10^ cells [21], and their storage function is regulated by reactive oxygen species (ROS), [22]. Incubation of platelets with MTs, decreasing the metabolically active labile pool of Zn^2+^, or injection of MTs into mice inhibits aggregation responses to collagen, probably due to the reduced phospholipase C (PLC)-mediated Ca^2+^ store mobilization and thromboxane A2 (TxA_2_) synthesis [23]. In addition, this led to the increased production of cyclic guanosine monophosphate (cGMP), thereby inducing antithrombotic effects in mesenteric venules [23]. Similarly, chelation of free ionic pool of Zn^2+^ with N,N,N′,N′-tetrakis 2-pyridinylmethyl-1,2-ethanediamine (TPEN) in the cytoplasm inhibits platelet aggregation and tyrosine phosphorylation [24], suggesting an important role of Zn^2+^ as a second messenger in platelet signaling.

The phospholipid membrane of eukaryotic cells is impermeable for Zn^2+^, therefore transport and storage of Zn^2+^ is ensured by Zn^2+^ transporters [2,8]. Resting platelets can uptake Zn^2+^ from the blood plasma and store it, indicating the existence of active Zn^2+^ transport and storage mechanisms [15,25,26]. Zn^2+^ concentration in blood serum was found to be higher than in blood plasma [27,28], suggesting that activated platelets could release a significant amount of Zn^2+^ during blood clotting. Later studies showed that incubation of platelets with extracellular Zn^2+^ increased the cytoplasmic and granular Zn^2+^ concentrations [29]. Hence, approximatively 50–80 pieces of α-granule fulfill the body of a single platelet [30,31,32], which possibly stores a significant amount of Zn^2+^. Therefore, it was proposed that the largest Zn^2+^ store could be located in platelet α-granules [26,29]. In addition, Zn^2+^ has a high affinity to bind fibrinogen, albumin, histidine-rich glycoprotein (HRG) and factor XIII, which are also accumulated in α-granules of platelets, suggesting that Zn^2+^ store exists as a protein-bound form in this type of granules [33]. Using inductively coupled plasma mass spectrometry (ICP-MS), Gorodetsky et al., reported that approximately 40% of Zn^2+^ is stored in granules, while 60% of Zn^2+^ is stored in other intracellular organelles and also in the cytoplasm [33]. Recently, the labile Zn^2+^ pool was visualized by our group and quantified in human and mouse platelets using a Zn^2+^ specific fluorescence dye FluoZin3 [26]. The specificity of the dye was characterized with Zn^2+^ supplementation or chelation of Zn^2+^ with ZnCl_2_ or TPEN, respectively. Using confocal microscopy, FluoZin3 staining showed several stained foci in the platelet cytoplasm, which became markedly reduced when platelets were spread on a fibrinogen-coated surface [26]. In addition, the rapid loss of intracellular FluoZin3 staining measured by flow cytometry, correlated to Zn^2+^ efflux in activated platelets [26]. Although α-granules are indicated as a major Zn^2+^ store in platelets [26,29], granule-located Zn^2+^ transporters and their functions in platelet signaling have not been investigated.

In mammalian cells, Zn^2+^ transporters are encoded by 24 different solute-linked carrier genes (*Slc30a/Slc39a*) and grouped into two protein families called ZIP and ZnT [16,34]. The *Slc39a* family was named ZIP (Zrt-, Irt-related protein), after the first two known members Irt1 of *Arabidopsis thaliana* and Zrt1 of *Saccharomyces cerevisiae*. So far, the exact structure of mammalian ZIP proteins has not been established, but 14 members of human and mouse ZIP isoforms and their genes have been isolated and characterized [16,17,35]. ZIP family members mediate Zn^2+^ influx from the extracellular place into cells, or from the intracellular Zn^2+^ store into the cytoplasm, thereby increasing cytoplasmic Zn^2+^ concentrations. Some ZIP isoforms are not strictly specific for Zn^2+^, and other metals such as iron (Fe), copper (Cu), cadmium (Cd), or manganese (Mn) could be also transported, depending on the pathophysiological status of the cell [2,8]. Recently, we showed mRNA expression levels of ZIP family members using in vitro-grown primary mouse MKs and quantitative real-time polymerase chain reactions (qRT-PCRs). Several isoforms of ZIP were predominantly expressed, including ZIP1, ZIP4, ZIP6, ZIP7, ZIP9 and ZIP10, while other ZIP isoforms showed limited mRNA expression, and ZIP2 was not detected [26]. Studies in mouse models showed the relevance of ZIP transporters in the regulation of systemic or cellular Zn^2+^ homeostasis [16,17,36], but the function of these transporters have been investigated neither in MKs nor in platelets. The members of *Slc30a* gene family, also called ZnT, are selective Zn^2+^ transporters located in the cell surface and on the membrane of intracellular organelles. So far 10 isoforms of ZnT have been identified in mammalians. ZnTs classically form homodimers and reduce Zn^2+^ levels using a H^+^/Zn^2+^ antiport mechanism. ZnT isoforms regulate Zn^2+^ efflux from the cytosol to the extracellular place or into intracellular organelles decreasing cytoplasmic Zn^2+^ concentration in mammalian cells [37]. Our studies showed that among ZnT isoforms, ZnT1, ZnT5, ZnT6, ZnT7 and ZnT9 are moderately or highly expressed in mouse MKs, while other ZnTs were weakly or not expressed [26].

**Figure 1:**
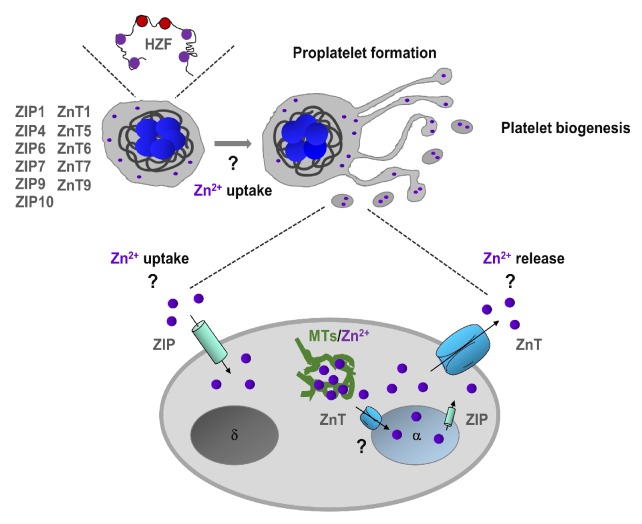
Zn^2+^ homeostasis in megakaryopoiesis and platelet biogenesis. Megakaryocytes express Zn^2+^-finger gene (Hzf), which regulates platelet formation and α-granule biogenesis. In addition, megakaryocytes express several ZIP (Zrt-, Irt-related protein)/Znt transporters which can regulate uptake and storage in α-granules. Extracellular and granule-resident Zn^2+^ may also regulate proplatelet formation and platelet biogenesis. In platelets, intracellular Zn^2+^ is mainly stored in α-granules, but it can also be sequestered by metallothioneins (MTs) in the cytoplasm. ZIP/Znt transporters are involved in Zn^2+^ homeostasis by regulating either efflux or influx of Zn^2+^ or both.

Hematopoietic Zn^2+^-finger gene (Hzf) is expressed mostly in megakaryocyte lineage [38]. Interestingly, modification of the zinc-finger domain of Hzf leads to impaired platelet formation and α-granule biogenesis [38]. Using FluoZin3, we have proposed that the intracellular Zn^2+^ store is located in α-granules of MKs [26], and therefore we speculate that Hzf function may be affected by the impaired granular Zn^2+^ store (Figure 1). In addition, Zn^2+^ also regulates the function of transcription factors GATA, which plays an essential role in megakaryopoiesis [39]. Although these studies indicate on potential Zn^2+^ homeostasis in MKs is very limited, the actual Zn^2+^ status regulates megakaryopoiesis and platelet biogenesis in mammalians needs further investigation.

## 3. Zn^2+^-Dependent Regulation of Platelet Function

The first link between Zn^2+^ and platelet function derives from the studies on rats submitted to experimental Zn^2+^ deficiency [40,41,42]. In these studies, Zn^2+^ deficiency resulted in prolonged bleeding time [40,42] and reduced platelet aggregation [41]. In other studies, using a similar experimental model, Zn^2+^ deficiency could impair platelet reactivity to agonists, such as adenosine diphosphate (ADP) and thrombin [43,44]. In addition, Zn^2+^ deficiency in rats is associated with impaired Ca^2+^ uptake of platelets, which can be normalized by in vitro incubation of blood with glutathione [45,46].

Bleeding abnormality associated with Zn^2+^ deficiency was also observed in human patients. In 1982, Gordon et al., showed that human volunteers submitted to experimental Zn^2+^ deficiency displayed defective platelet aggregation response to ADP and arachidonate, and Zn^2+^ supplementation could normalize these defects [47]. Later, ecchymosis, abnormally prolonged bleeding and platelet aggregation responses were described in two cancer patients, one with squamous cell carcinoma and another with non-Hodgkin’s lymphoma, with concurrent nutritional Zn^2+^ deficiency [48].

Recently, it has been proposed that intracellular chelation of Zn^2+^ in platelets can inhibit tyrosine phosphorylation cascades, thereby attenuating platelet reactivity and aggregation responses in vitro [24]. On the other hand, it has been shown that increased levels of dietary Zn^2+^ in rats correlates with enhanced platelet responses to suboptimal doses of collagen, ADP, thrombin and epinephrine, suggesting the link between actual Zn^2+^ status and platelet reactivity [49]. Zn^2+^-mediated platelet aggregation is enhanced [49], because fibrinogen binding to the integrin αIIbβ_3_ was enhanced and consequently increasing the binding capacity of platelets to other blood cells and also to the fibrin clot.

Receptor-mediated activation of phospholipase C (PLC) is essential to increase the activity of diacylglycerol (DAG)-sensitive Ca^2+^ channel transient receptor potential cation channel subfamily C member 6 (TRPC6), and to induce Ca^2+^ store depletion through inositol 1,4,5-trisphosphate (IP_3_)-mediated activation of IP_3_-receptor (IP_3_R), which further modulates store-operated Ca^2+^ entry (SOCE) by activation of the stromal interaction molecule 1 (STIM1)–Orai1 complex in platelets [50]. Interestingly, Zn^2+^ status of the cells is connected to Ca^2+^ homeostasis and regulates the actual cytoplasmic Ca^2+^ levels as well, thereby indirectly influencing platelet reactivity. Indeed, when rats were fed with a low Zn^2+^ diet, increased basal Ca^2+^ concentration at resting stage was observed, but surprisingly, Ca^2+^ influx was significantly reduced without any change on Ca^2+^ store depletion, indicating that platelet Zn^2+^ deficiency impairs Ca^2+^ channel function, independently of SOCE mechanism [46]. However, in sharp contrast, 50% reduction of IP_3_ production was observed in platelets isolated from Zn^2+^-deficient rats, when Zn^2+^ concentration was at least 70% lower in the blood plasma than controls [51], suggesting that depending on the severity of Zn^2+^ deficiency, Zn^2+^ could also influence Ca^2+^ store depletion and SOCE. However, the molecular mechanisms of Zn^2+^-dependent SOCE and the physiological crosstalk between Zn^2+^-dependent signaling and Ca^2+^ channels has not been established. Interestingly, protein kinase C (PKC), the downstream effector of PLC, directly binds Zn^2+^ [52]. Subcellular localization of PKC is modified by increased levels of Zn^2+^ in the cytoplasm; accumulation of activated PKC was detected close to the plasma membrane of platelets. On the other hand, a PKC antagonist GF109203X could strongly inhibit Zn^2+^-mediated platelet aggregation responses [24], indicating a complex role of PKC in Zn^2+^ and Ca^2+^-mediated signaling pathways.

Zn^2+^ binds to sulfhydryl groups and protects several signaling molecules and channels from oxidation, thereby regulating ROS-mediated signaling of the cells [53]. Reduced intracellular Zn^2+^ levels can change the biochemical properties of signaling molecules and the activity of cation channels by increasing oxidation of free sulfhydryl groups, thereby modifying the structure of these proteins. Interestingly, TRPC6 is directly activated by ROS [54], while transient receptor potential melastatin channel 2 (TRPM2) is indirectly activated, which further enhances the channel activity in MKs [55]. Although there are other TRPM channels, transient receptor potential melastatin channel 7 (TRPM7) and the Orai1 channel are also redox-sensitive, and their channel activity is inhibited by ROS [56,57]. In addition, it has been shown that TRPC6 and TRPM7 can transport both Zn^2+^ and Ca^2+^ into the cells [58,59,60], but their functions in Zn^2+^ homeostasis have not been investigated in MKs and platelets. Therefore, future studies are necessary to elucidate the function of these channels in the regulation of Zn^2+^ and Ca^2+^ homeostasis, which are probably connected by PKC and ROS in MKs and platelets.

## 4. Zn^2+^-Dependent Hemostasis and Fibrin Clot Formation

Platelets cannot synthesize fibrinogen, it is produced and released by liver hepatocytes into the blood plasma and taken up and stored in α-granules of circulating platelets [61]. Fibrinogen is composed of two pairs of three disulfide-linked polypeptide chains [62,63]. These chains are symmetrically folded and composed of a central E-domain with two lateral D-globular domains. Thrombin activation is an essential step of the hemostatic process, regulating the coagulant activity, and converting the soluble fibrinogen to insoluble fibrin fibers thereby stabilizing the growing blood clot [64]. Fibrin assembly is initiated after thrombin-induced removal of fibrinopeptide A from plasma fibrinogen. Several steps of thrombin proteolysis on fibrinogen create an active fibrin monomer, which is polymerized subsequently to fibrin protofibrils. Lateral assembly and covalent linkage of hundreds of protofibrils could form the fibrin fiber [62,65]. Irregular assembly of fibrin fibers finally forms the fibrin network within the blood clot. The fibrin clot is composed of different diameters and lengths of fibrin fibers. Depending on the ratio between thin and thick fibers, which is regulated by thrombin, Zn^2+^, Ca^2+^ and other factors, fibrin clot structure and mechanical stability can be different [62,66]. When fibrin clot is mainly composed of thin and densely packed fibers, fibrin clot is less porous and stiffer, thereby increasing their potential for rupture and embolism. When thick fibers are dominant within the fibrin clot, it is less stiff, more porous; therefore, a fibrin clot can change easily the size and form, depending on the actual shear stress [62]. Several studies showed the multiple effects of Zn^2+^ and Ca^2+^ on fibrin clot formation because fibrinogen contains several binding sites for both cations [66,67]. Binding of fibrinogen to Ca^2+^ enhances the kinetics of fibrin clot formation and also modifies the structure of the fibrin network; more thick fibers were polymerized [68]. Although it is well established that Ca^2+^ plays an important role in different steps of fibrin clot formation, Ca^2+^-dependent mechanism behind this process has been published controversial [69,70].

Initial studies showed that Zn^2+^ can inhibit amidolytic activity of thrombin [71,72] and diminishes thrombin-mediated fibrinopeptide A release [73]. Studies by Marx also demonstrated that Zn^2+^ can bind fibrinogen and fibrin with Kd values ranging from 8 to 18 µM and with a ratio of six Zn^2+^ atoms per fibrin/fibrinogen molecule [66]. Interestingly, binding sites of Zn^2+^ are mapped in the lateral D-globular domains, but not in the central E-domain and sites are distinct from those of Ca^2+^ [74,75,76], indicating an alternative regulatory mechanism of fibrin formation. Similar to Ca^2+^, Zn^2+^ also accelerates the rate of thrombin-induced fibrin clot formation, whereas this effect was not observed in the presence of magnesium (Mg^2+^) or manganese (Mn^2+^) [66,67], suggesting that modulation of thrombin-induced fibrin formation is specific to Zn^2+^ and Ca^2+^. It has been shown that the lateral association of fibrin monomers to protofibrils is enhanced by Zn^2+^ to a greater extent than Ca^2+^, and Zn^2+^ induces a fibrin clot that has reduced fiber stiffness, compared to Ca^2+^-induced fibrin clot [66,75,77,78]. Interestingly, on the one hand Zn^2+^ attenuates the activity of thrombin but on the other hand can still enhance fibrin clot formation. This may indicate that Zn^2+^ enhances clot formation likely through a mechanism potentiating tridimensional assembly of fibrin network.

Recently, we demonstrated that lack of α-granule content strongly influences the structure of the fibrin network in vitro [26]. Releasate of wild-type and *Nbeal2^-/-^* platelets were observed by scanning electron microscopy (SEM) and ultrastructure of fibrin clot was obviously different and fibrin structure was partially modified in the presence of extracellular Zn^2+^, indicating an important role of α-granule-resident Zn^2+^ and fibrinogen release during fibrin clot formation [26].

**Figure 2:**
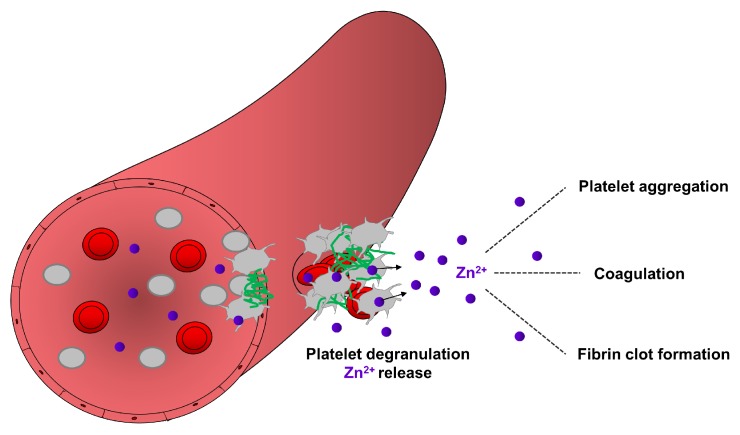
Contribution of platelet-resident Zn^2+^ in the regulation of hemostasis. During hemostasis, a process which stops bleeding, platelets accumulate in the site of injury and became activated. Activated platelets release Zn^2+^ from their granules, which may enhance the aggregation of circulating platelets, modulate the activity of proteins of coagulation and fibrinolytic pathways. Zn^2+^ can bind to fibrinogen and fibrin and modulate the ultrastructure and mechanical strength of fibrin, thereby supporting fibrin clot formation during hemostasis.

Fibrin formation is enhanced by the release of extrinsic factors from the damaged vessel wall or intrinsic factors from blood cells and platelets [62,68]. The intrinsic pathway of coagulation is initiated by activation of factor XII which further modulates the kininogen/kallikrein system, referred to as the plasma contact system. Polyphosphate accumulation on the surface (also called polyanionic surface) of endothelial cells and platelets triggers the activation of factor XII which further activates its substrate factor XI, thereby activating fibrin formation [79,80]. On the other hand, factor XII also induces the pro-inflammatory cascade through the liberation of the inflammatory peptide hormone bradykinin by kallikrein-mediated cleavage of kininogen [80,81]. Accumulation of bradykinin on the endothelial cell surface increases vascular permeability, promoting adhesion of inflammatory immune cells [82,83,84]. Zn^2+^ is also known to regulate several steps of the coagulation cascade to activate plasma factors and the contact system [25,85]. Zn^2+^ directly binds to factor XII, inducing a conformational change, thereby improving its susceptibility for enzymatic activation [86]. Moreover in the presence of Zn^2+^, factor XI binds to glycocalicin (the soluble extracellular region of GPIbα), thereby affecting the thrombin generation pathway [87]. The activated form of factor XI enhances factor IX activity and the formation of the tenase complex, thereby further promoting thrombin activation and fibrin formation, [25,83]. Consequently, Zn^2+^ deficiency could strongly impair the coagulation cascade and fibrin formation leading to prolonged bleeding times, as already demonstrated in rodent models and human patients [15]. Together these studies suggest that Zn^2+^ may contribute to hemostasis through several mechanisms modulating platelet aggregation, coagulation and fibrin network formation (Figure 2).

## 5. Zn^2+^ in Thrombosis

**Figure 3:**
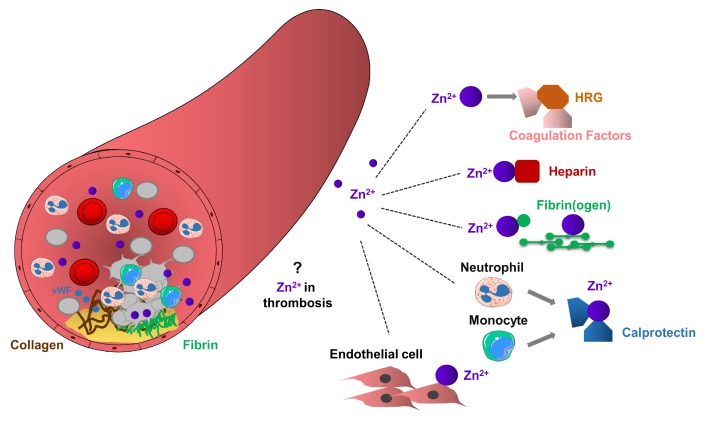
Potential role of Zn^2+^ in arterial thrombosis. Arterial thrombosis occurs in atherosclerotic plaque rupture, as a result of clot formation, leading to platelet aggregation, thrombus formation and vessel occlusion. Platelets through glycoproteins, interact with collagen or von Willebrand Factor (vWF), change their shape and adhere to the site of injury. Adherent platelets become activated and release their granule content leading to the recruitment of additional circulating platelets. Extracellular Zn^2+^ or Zn^2+^ released from platelet granules may enhance thrombosis by mediating interactions between HRG and coagulation factors, Zn^2+^ sequesters heparin, or interact with fibrinogen and fibrin, thereby enhancing fibrin deposition and stimulating recruitment of circulating platelets and subsequent thrombus growth. Calprotectin released from neutrophils and monocytes may interact with Zn^2+^ and consequently enhance prothrombotic events. Procoagulant endothelial cells may provide favorable surface to the accumulation of Zn^2+^, thereby enhancing thrombosis.

Platelet accumulation and activation at the sites of vascular injury lead to the release of granular located Zn^2+^ from platelets to the microenvironment of the vascular network [25]. Several indirect experimental pieces of evidence support the role of platelet Zn^2+^ in thrombosis, but the molecular mechanisms of how platelet-resident Zn^2+^ may modulate this process has not been established. It is possible that activated platelets can increase the local concentration of Zn^2+^ during thrombosis, enhancing fibrin deposition and subsequent recruitment of additional circulating platelets, thereby potentiating thrombus growth. Zn^2+^ released by platelets may act as a co-factor for initiating the assembly of the contact system on the polyanionic surfaces and also on the procoagulant surface of platelets and endothelial cells during thrombosis [15,25,88,89], (Figure 3).

HRG is important regulator in the coagulation cascade since they can directly bind Zn^2+^, heparin, plasminogen, fibrinogen and other factors in the blood plasma [90]. In addition, interactions between HRG and many of these proteins are stimulated by Zn^2+^ [25]. High levels of HRG are associated with the clinical symptoms of various cardiovascular disorders, while congenital deficiency of HRG is connected to thrombophilia in humans [91,92]. Interestingly, free fatty acids (FFAs) allosterically disrupt Zn^2+^ binding to albumin, indicating that increased FFA content could modulate the dissociation of Zn^2+^ from albumin, thereby increasing the concentrations of metabolically active Zn^2+^ pool in the blood [93,94]. Consequently, high levels of FFA in patients with cancer, diabetes or obesity may shift the Zn^2+^ pool from albumin to HRG, thereby increasing the risk of thrombosis [93]. In addition, Zn^2+^ may regulate the adhesion of immune cells through the ability to bind the plasma HRG [90].

Calprotectin (a complex of S100A8 and S100A9) is one of the major Ca^2+^ and Zn^2+^ binding proteins. Calprotectin can sequester Zn^2+^, reducing the metabolically active labile Zn^2+^ pool in the blood, thereby limiting Zn^2+^ uptake by microbes, as a process termed nutritional immunity [95]. Under inflammatory conditions, calprotectin is released from activated neutrophils and monocytes to the bloodstream [95]. Therefore, the permanent increase of plasma concentrations of calprotectin can induce hyperzincaemia in human patients, characterized by recurrent infections, hepatosplenomegaly, anemia and chronic systemic inflammation, and in addition, presenting dermal ulcers, cutaneous inflammation and spontaneous hematomas [96]. Moreover, in patients with coronary artery diseases, high levels of calprotectin were associated with a reduced effect of aspirin, as indicated by increased platelet aggregation [97,98]. Although platelets can store and release calprotectin [98,99], the function of platelet-resident calprotectin in hyperzincaemia induced by chronic inflammation has not been investigated.

## 6. Zn^2+^ in Ischemic Stroke

Aberrant Zn^2+^ accumulation in the brain was observed in various neurological disorders, including traumatic brain injury, stroke and seizure [100,101]. Ischemic stroke is known as thrombo-inflammatory diseases involving the recruitment of platelets and immune cells to the site of ischemic vascular injury, damaging the permeability of the blood-brain barrier (BBB), thereby triggering neuronal cell death [50]. BBB and the blood-cerebrospinal fluid barriers maintain Zn^2+^ transport in the brain. In animal models of transient global and forebrain ischemia, accumulation of Zn^2+^ was observed in neuronal tissue at a late step of ischemic insult, before the onset of cellular death [102]. After 3 h of mild transient focal ischemia, Zn^2+^ accumulation in cortical neurons was also obvious, thus accelerating the development of cerebral infarction [102]. In addition, aberrant excess of Zn^2+^ into the synaptic cleft of ischemic neurons was found to induce toxicity, therefore Zn^2+^ can be an independent risk factor for ischemic stroke [102]. Consequently, administration of the Zn^2+^ chelator, di-calcium ethylene diamine tetra-acetic acid (Ca_2_EDTA) in rats subjected to experimental ischemia resulted in a neuroprotective effect. Ca_2_EDTA could inhibit neuronal damage by reducing the brain infarct size, improves neurological function and prevents apoptosis in ischemic neurons [102]. Furthermore, a selective Zn^2+^ and Ca^2+^ chelator DP-b99 also has neuroprotective properties in animal models and also in patients with acute ischemic stroke [103,104,105]. It has also been shown that copper/zinc superoxide dismutase could attenuate neuronal death and hippocampal injury after transient focal and global ischemia [106,107]. In rats, systemic administration of Zn^2+^ protects hippocampus from neuronal damage during the reperfusion phase of transient cerebral ischemia [108]. Patients with ischemic stroke frequently have a lower dietary Zn^2+^ intake (50% of the recommended amount) during hospitalization, and after normalization of Zn^2+^ intake, patients have better recovery of neurological deficits [109]. These conflicting results suggest that Zn^2+^ has multiple roles in the early and late phases of stroke development, and the observed positive or negative effects described here are strongly dependent on the in vivo experimental conditions.

In neurons, 10% of total Zn^2+^ content is metabolically active Zn^2+^ which is mainly located in presynaptic vesicles of glutamatergic nerve terminals and acts as a second messenger on the postsynaptic neuron during degranulation [110]. Following ischemic brain insult, Zn^2+^ is released together with glutamate into the synaptic cleft, and depending on the activating stimulus, Zn^2+^ concentration can be increased up to 100 µM [111]. In pathological conditions, glutamate-induced Ca^2+^ overload is a major cause of neuronal death, but besides this process, abnormal accumulation of Zn^2+^ can also act on the postsynaptic membrane in a synergistic mode with Ca^2+^. Indeed, N-methyl-D-aspartate receptors (NMDARs), voltage-sensitive Ca^2+^ channels (VSCCs), or a cation-permeable form of α-amino-3-hydroxy-5-methyl-4-isoxazolepropionic acid (AMPA) receptors on the postsynaptic membrane can regulate both Zn^2+^ and Ca^2+^ uptake mechanisms [112]. Consequently, pharmacological inhibition of glutamate receptor 2 (GluR2)-lacking AMPA receptor reduces the Zn^2+^ level in the cytoplasm and, therefore, neurons become protective in the ischemic brain [113].

Neuroprotective strategies of ischemic stroke were strongly focused on the blockade of aberrant Ca^2+^ influx-mediated by the intracellular Ca^2+^ store and SOCE [50,114,115]. Inhibition of neuronal SOCE in mouse models of a stroke, regulated by stromal interaction molecule (STIM) 2 and ORAI2, was proposed to be an alternative route to avoid Ca^2+^ overload in ischemic neurons [116]. Interestingly, glutamate-induced Ca^2+^ overload leads to acidification of the cytoplasm, which modulates the MT-resident Zn^2+^ store, thereby further increasing the concentration of the labile Zn^2+^ pool, inducing cytotoxicity of ischemic neurons [117]. Consequently, blockade of SOCE in ischemic neurons may inhibit both the Ca^2+^ overload and aberrant Zn^2+^ response from the intracellular Zn^2+^ store regulated by MTs.

Beside Ca^2+^ and Mg^2+^, TRPM7 channel can also transport Zn^2+^ and regulate systemic and cellular Zn^2+^ homeostasis in mice [118]. Several in vivo studies demonstrated that TRPM7 function contributes to ischemic stroke [119], although the involvement of its channel and kinase activity in the regulation of Zn^2+^ homeostasis under ischemic conditions is still under investigation. Recently, we demonstrated that the kinase activity of TRPM7 is a key modulator of SOCE in platelets, and this signaling pathway was confirmed in other cell types, suggesting an interplay between the kinase domain of TRPM7 and STIM1 [120]. Consequently, deletion of TRPM7 kinase activity in mice significantly reduces SOCE in platelets, and protected mice from ischemic brain insults, indicating that the increased kinase function, rather than its channel function of TRPM7, plays an important role in ischemic stroke [120]. Furthermore, impaired channel activity of TRPM7 induces macrothrombocytopenia in mice and humans [121], implicating that long-term inhibition of TRPM7 channel activity in disease condition may alter platelet biogenesis and function. Therefore, an alternative strategy to develop safe drugs against TRPM7 function in stroke would be a selective targeting of the kinase activity of TRPM7, thereby inhibiting thrombo-inflammation and protecting BBB and neurons from ischemic insults.

So far, the function of Zn^2+^ transporters in stroke development has not been established, and only a few experimental studies determined the expression profile of Zn^2+^ transporters. Tsuda and colleagues examined the induction of ZnT1 mRNA expression in the CA1 subfield of the hippocampus following global ischemia [122]. It was postulated that in response to the increased levels of intracellular Zn^2+^, vulnerable neurons would upregulate ZnT1, to facilitate Zn^2+^ efflux in the plasma membrane. While ZnT1 mRNA expression was enhanced as soon as 12 h post-ischemia, without subsequent ZnT1 protein expression, cellular demise ensured by three days post-ischemia [122]. The expression levels of ZnT1 and ZnT6 transporters were increased in the mouse model of hippocampal damage-induced by hypobaric hypoxia. Moreover, chelation of Zn^2+^ with Ca_2_EDTA decreased the expression of ZIP6 transporter and attenuated the hypobaric hypoxia-induced oxidative stress, inflammation and apoptosis [123].

The present therapy for acute ischemic stroke is limited to thrombolysis with the recombinant tissue plasminogen activator (rtPA) and mechanical recanalization [124]. Therefore, a better understanding of the molecular interplay between Zn^2+^ and Ca^2+^ homeostasis in ischemic stroke is needed for the development of more effective therapies. Future studies are needed to address the regulatory mechanisms, whether platelet-resident Zn^2+^ may directly regulate stroke or other thrombo-inflammatory diseases.

## 7. Dysregulated Zn^2+^ Homeostasis in Storage Pool Diseases

Secretory granule release is critical for platelet activation and hemostasis. Human platelets characterized by the lack or defective function of secretory granules show impaired aggregation responses, but depending on a genetic disorder, only exhibits minor to moderate bleeding [125]. Storage pool disease (SPD) comprises two groups of genetic disorders in which the function of platelet α and/or δ granules is defective [126]. In humans and mice, mutations in the *Nbeal2* gene results in gray platelet syndrome (GPS), a rare inherited bleeding disorder characterized by thrombocytopenia, lack of α-granules within blood platelets and progressive development of bone marrow fibrosis [127,128,129,130]. NBEAL2 function was proposed to regulate the sorting and packaging processes of α-granules in MKs [31], only large empty vacuoles could be detected in the platelet cytoplasm of GPS patients, α-granule content is very limited or missing, and the function of residual granule-like structures is defective [131]. Consequently, most of the α-granule-resident proteins are degraded in the lysosomes, although P-selectin (as a marker of α-granules) could be detected on the platelet surface [131]. The lack of α-granules in GPS platelets strongly reduces fibrinogen content, but interestingly, fibrinogen uptake is not defective, thus a limited amount of fibrinogen can be stored and secreted by GPS platelets [132]. It is important to note that δ-granule biogenesis and function are normal in GPS patients, therefore ADP, serotonin and cations can be released upon platelet activation, maintaining the feedforward loop of platelet activation. Nevertheless, the lack of α-granules and degradation of α-granule-resident proteins, including plasma factors and fibrinogen, possibly impair fibrin clot formation in GPS patients. Recently, we investigated whether defective granule biogenesis or secretion may impair the platelet Zn^2+^ store and release, thereby influencing fibrin clot formation [26]. For this study, a turbidity assay was performed to quantify the thrombin-induced fibrin formation in wild-type mice and also in a mouse model of GPS (*Nbeal2^-/-^* mice). We found that turbidity was lower in wild-type platelet-releasate, which was further decreased in the presence of extracellular Zn^2+^. In *Nbeal2^-/-^* mice turbidity was similar to resting level in platelet releasate after thrombin activation, but it was significantly reduced in the presence of Zn^2+^ [26]. However, extracellular Zn^2+^ cannot fully restore fibrin formation to the wild-type level, due to the strongly reduced fibrinogen content and release from α-granules of *Nbeal2^-/-^* platelets [26]. Using microfluidics, we measured the kinetics of fibrin clot formation and calculated the accumulation rate of fluorescently-labeled platelets and fibrin fibers. Zn^2+^ supplementation did not modify the kinetics of fibrin formation in wild-type blood, suggesting that extracellular Zn^2+^ seems to be not a limiting factor of blood plasma in healthy conditions [26]. However, in disease conditions, platelet-dependent fibrin clot formation under flow was impaired in whole blood samples from *Nbeal2^-/-^* mice, and the kinetics of fibrin formation was accelerated by Zn^2+^ supplementation [26]. These alterations may also point to an impaired Zn^2+^ transport into the available secretory granules, dysregulating cytoplasmic Zn^2+^ levels in platelets. Taken these results together, we propose that platelets from GPS mouse model and human patients have a strongly reduced Zn^2+^ store, which influences platelet Zn^2+^ homeostasis and fibrin clot formation. Limited Zn^2+^ efflux during degranulation of GPS platelets, in addition to reduced fibrinogen release, could also inhibit fibrin formation and the activation of coagulation cascade. However, further investigation is necessary to understand the exact role of platelet Zn^2+^ release in these pathological conditions.

Prolonged bleeding time is frequently measured in SPD patients, which is usually ascribed with reduced ADP and serotonin content and release. However, the genetic background of SPD disease with δ-granule deficiency is still under debate. Hermansky–Pudlak Syndrome (HPS) is phenotypically associated with malformation or reduced numbers of platelet δ-granules and a bleeding diathesis [125,131], but α-granule content and function are unaffected, and therefore platelet activation is only mildly or moderately defective. Depending on the genetic mutation, HPS patients are divided into several subtypes, HPS-1 is the most common form characterized by hypopigmentation and albinism. In clinical treatment, antifibrinolytic agents have been administrated to these patients to prevent bleeding complications [126]. Using FluoZin3 measurement, our preliminary data showed impaired basal Zn^2+^ levels in HPS and SPD patients, indicating defective Zn^2+^ homeostasis in the granular store of these patients [26]. However, we assume that platelet responses to Zn^2+^ and also fibrin formation could be different, depending on the genetic background of the HPS/SPD patients and the severity of granule release. In the mouse model, platelets lacking the putative vesicle docking protein Munc13–4 (*Unc13d^-/-^*) have a completely abolished δ-granule secretion [133]. The lack of the feedforward loop induced by second wave mediators (ADP, serotonin) located in δ-granule strongly impairs α-granule release in *Unc13d^-/-^*platelets as well, which resulted in platelet dysfunction and severely prolonged tail-bleeding time [133], probably due to the combined defects of ADP/serotonin release from δ-granules and α-granule-resident proteins vWF, fibrinogen and coagulation factors. Using flow chamber assay, we showed that platelet-dependent fibrin clot formation was impaired in whole blood samples of mutant mice, and the kinetics of fibrin formation was accelerated by Zn^2+^ supplementation [26].

## 8. Conclusions

Taking into account these findings from the limited number of patients and mouse models with granule storage deficiency or granule release defects, it worth postulating that Zn^2+^ homeostasis could be strongly impaired in patients with platelet granular abnormalities. Consequently, reduced α-granule content or blockade of α-granule secretion can impair the coagulation pathway and fibrin clot formation in humans, which can be more critical in patients with combined defects of both α and δ-granules or with thrombocytopenia. In future, Zn^2+^ supplementation could be used in mouse models of GPS and SPD, to manipulate the size and structure of fibrin fibers and study fibrin clot formation under static and shear conditions, to allow for a better understanding of Zn^2+^-dependent clot stability and bleeding tendency in these pathological conditions. In addition, future studies are needed to identify the subcellular localization of platelet-specific Zn^2+^ transporters as a basis for understanding the molecular mechanisms of platelet Zn^2+^ uptake, storage and release and contribution of ZIP/ZnT isoforms to Zn^2+^ homeostasis. Whether the expression profiles of ZIP/ZnT isoforms are changing during megakaryopoiesis in normal and disease conditions, and which ZIP/ZnT isoforms regulate basic metabolic pathways between the Zn^2+^ store and platelet cytoplasm, which are impaired in GPS/SPD platelets, still needs to be further investigated at mRNA and protein levels. Future studies using mouse models with ZIP/ZnT deficiency in megakaryocyte/platelet lineage are needed to investigate the role of Zn^2+^ transport and storage in megakaryocytes and platelet-related diseases, including thrombosis and ischemic stroke.
